# The Invasive Blue Crab *Callinectes sapidus* from the Northern Adriatic Sea: Feeding Behaviour and Fecundity Data

**DOI:** 10.3390/ani16111576

**Published:** 2026-05-22

**Authors:** Antonio Casalini, Laura Gentile, Dario Lombardi, Riccardo Brusa, Pietro Emmanuele, Oliviero Mordenti

**Affiliations:** Department of Veterinary Medical Sciences, Alma Mater Studiorum University of Bologna, 40064 Ozzano dell’Emilia, BO, Italy; antonio.casalini6@unibo.it (A.C.); dario.lombardi4@unibo.it (D.L.); riccardo.brusa@studio.unibo.it (R.B.); pietro.emmanuele@unibo.it (P.E.); oliviero.mordenti@unibo.it (O.M.)

**Keywords:** blue crab, prey selection, reproductive potential, cannibalism, feeding behaviour

## Abstract

The population of the blue crab *Callinectes sapidus* is rapidly expanding in the Mediterranean Sea, where it is causing increasing ecological and economic impacts. However, biological information from recently colonised areas remains limited. This study combined controlled experiments and reproductive analyses to investigate the key biological traits of *C. sapidus* in the northern Adriatic Sea. Feeding trials were conducted under controlled conditions to assess prey consumption and feeding behaviours using Manila clams (*Ruditapes philippinarum*) and Mediterranean mussels (*Mytilus galloprovincialis*), offered as monospecific and mixed diets. The results showed that, while the number of prey items consumed did not differ markedly between species, mussels provided a significantly higher edible biomass, indicating greater energetic profitability. Handling and consumption times were comparable between prey types, suggesting compensatory differences between opening and feeding phases. Food intake was strongly influenced by temperature, with peak consumption observed between 25 and 28 °C and reduced feeding at both lower and higher temperatures. Reproductive analysis of ovigerous females revealed high fecundity, with a mean of 1.63 ± 0.53 million eggs per individual and a positive relationship between fecundity and body size. Overall, the results confirm that *C. sapidus* combines trophic flexibility with high reproductive output—traits that support its invasive success. These findings improve the understanding of the blue crab’s ecological role in the northern Adriatic, providing useful insights for assessing its impact on shellfish aquaculture and developing management strategies.

## Simple Summary

*Callinectes sapidus* (blue crab) is an invasive species that has rapidly spread in the Mediterranean Sea, including the northern Adriatic, where it is causing ecological and economic problems. It preys on commercially important shellfish such as clams and mussels. This study investigated two key aspects of the blue crab’s biology: its feeding behaviour and its reproductive potential. Feeding experiments carried out under controlled conditions showed that blue crabs could consume both clams and mussels efficiently. Although they sometimes ate a greater number of clams, mussels provided a larger amount of edible biomass, suggesting that prey choice may depend on the balance between energetic gain and the effort required to open and consume the prey. Temperature also influenced feeding activity, with the highest consumption observed between 25 and 28 °C. The reproductive analysis revealed that female crabs produce a very high number of eggs—more than 1.6 million per brood, on average—and that larger females produce more eggs. These results show that the blue crab combines high feeding flexibility with strong reproductive capacity, characteristics that help to explain its rapid expansion. Understanding these traits is essential for assessing the blue crab’s potential impacts on ecosystems and shellfish aquaculture, as well as for supporting future management strategies.

## 1. Introduction

*Callinectes sapidus* (blue crab) is recognised among the 100 worst invasive alien species in the Mediterranean Sea [1]. In Italian coastal waters, its invasion history extends from the first record in the Grado Lagoon in 1949 to its current broad distribution across the northern Adriatic, the central and southern Adriatic, the Ionian Sea, and Sicilian coastal systems [2,3,4,5]. This rapid expansion reflects the species’ remarkable ecological success in Mediterranean coastal environments and highlights the need to better understand the biological traits supporting its establishment, persistence, and spread [3,4,5].

The invasion success of *C. sapidus* is strongly associated with its physiological and ecological plasticity [4]. This species is both euryhaline and eurythermal, tolerating broad environmental fluctuations, and inhabits estuaries, lagoons, and coastal habitats over a wide salinity and temperature range [4,6,7]. This tolerance enables the colonisation of highly variable transitional systems, including shallow lagoon environments that are often brackish, eutrophic, and thermally dynamic [4,8,9]. Such habitats are particularly favourable for the species and may enhance the expression of traits linked to invasion success, including trophic flexibility, aggressive behaviour, and high reproductive output [4,8,9].

Reproductive potential is one of the key life-history traits underlying the spread of the blue crab [3,10,11,12]. Females can produce large egg masses, fecundity generally increases with body size, and multiple broods may be produced over the reproductive lifespan, resulting in a high cumulative reproductive output [10,11,12]. In invaded Mediterranean systems, recent studies have also documented prolonged reproductive activity, often spanning from late spring to early autumn, with a prolonged presence of ovigerous females and high fecundity, all of which may accelerate population growth and favour rapid establishment in newly colonised areas [6,11,13]. These reproductive traits are central to the invasion dynamics of *C. sapidus*, and deserve particular attention in areas where the species is still expanding [3,6,11,13].

Another defining trait of *C. sapidus* is its trophic versatility [4,6,14]. The species is widely described as an opportunistic and aggressive omnivorous predator capable of exploiting a broad spectrum of prey, including molluscs, crustaceans, fish, polychaetes, and other benthic resources. It may also display scavenging and cannibalistic behaviour, confirming a wide trophic niche and strong dietary flexibility [4,6,14]. In Italian coastal waters, this trophic plasticity contributes substantially to the ecological and socioeconomic relevance of its invasion, as the species can affect local food webs, prey upon native and farmed species, and generate negative consequences for small-scale fisheries and aquaculture [3,15].

Predation on commercially important bivalves is one of the most direct and economically significant threats in the northern Adriatic [7,8,16,17]. Clam and mussel production systems may experience substantial losses, while broader socioeconomic consequences may affect shellfish farmers, fisheries, and the food value of coastal resources [7,17]. Experimental studies have shown that *C. sapidus* is an efficient bivalve predator with broad prey breadth and that predation outcomes depend on prey identity and prey size as well as ecological context, including substrate characteristics and prey burial capacity [8,18,19,20]. As such, the northern Adriatic represents a crucial case study, given that the species’ recent expansion overlaps with highly productive lagoon environments and major shellfish-farming areas [7,8,16,17].

Beyond direct predation on commercial species, the invasion of *C. sapidus* may also produce wider ecosystem-level effects, including alterations in benthic trophic structure, community composition, and ecological interactions with other non-indigenous species [3,21]. At the broader Mediterranean scale, its invasion dynamics appear to be associated with climate-related environmental change and nutrient enrichment in shallow coastal systems, suggesting that ecological impacts may be modulated by ongoing environmental change [5,22]. This combination of ecological plasticity, trophic opportunism, and high reproductive capacity makes the blue crab a particularly challenging invasive species from both biological and management perspectives [3,5,9,22].

Despite the growing attention paid to the species in the Mediterranean basin, biological information from recently colonised areas remains incomplete, especially when trophic and reproductive traits are considered together within the same local context [3,6,8]. Previous studies have documented important aspects of its feeding ecology, prey use, thermal response, and reproductive biology, but these dimensions are often addressed separately [6,8,9,11,13,20]. Consequently, the functional linkage between resource exploitation, environmental modulation of feeding activity, and reproductive output remains insufficiently resolved in newly invaded systems, particularly in areas where ecological impacts and aquaculture conflicts are rapidly emerging [7,8,16,17].

Within this broader ecological and socioeconomic context, this study investigated complementary biological traits of *C. sapidus* that are relevant to its invasive success in the northern Adriatic. This study combined the following: (i) experimental assessment of prey use and prey profitability on commercially relevant bivalves; (ii) an evaluation of prey handling and consumption time; (iii) an analysis of temperature-related variation in feeding activity; and (iv) reproductive data from ovigerous females collected in the same regional context. This study integrated these complementary trophic and reproductive perspectives, aiming to provide a more coherent biological framework for interpreting the ecological impacts of the blue crab and its implications for shellfish aquaculture and invasion dynamics in a recently colonised Mediterranean environment.

## 2. Materials and Methods

*Sampling*

**Figure 1:**
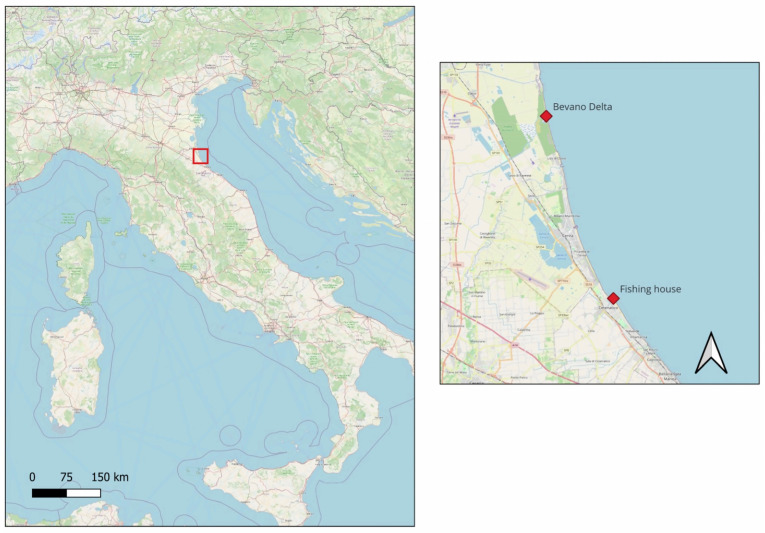
Location of the sampling sites. Bevano Delta and fishing house indicate the two capture sites where individuals were collected for this study.

Animals were caught in the Bevano River Delta (44°21′41.94″ N, 12°19′26.45″ E) in August 2025. The Bevano River Delta is a protected natural area on the northern Adriatic coast of Italy and is part of the Po River Delta Park. In addition, blue crabs were also collected by the *Cooperativa Pescatori Laghese* (Lagosanto, FE) during the same period. Another group of animals, obtained from a fishing house in Cesenatico in August 2025 (44°12′28″ N, 12°24′11″ E), was used in reproduction studies (Figure 1).

Crabs were captured using trammel nets, which comprised three layers of netting, with two different mesh sizes: the two outer layers had larger meshes, while the inner layer had a smaller mesh. The caught animals (n = 250) were transported in an insulated tank (5.50 m^3^) to the Veterinary Medical Sciences Department of the University of Bologna’s ichthyological greenhouse, where they were acclimatised for 7 days under controlled conditions in the RAS, maintaining the same water parameters used during the trials (a temperature of 23 ± 1 °C, a dissolved oxygen level of 7.0 ± 0.5 mg L^−1^, pH 8.2 ± 0.1, and a salinity level of 23 ± 1 PSU). They were not fed during this period, in order to standardise their physiological conditions.

*Morphometrics*

The crabs were anaesthetised using 0.4 mL/L of phenoxyethanol to minimise stress during handling. Following anaesthesia, the crabs were sexed, and then their carapace length (CL), carapace width (CW), and body weight (BW) were measured using a digital calliper (±0.01 mm precision) and recorded. CW was measured between the outermost points of the lateral spines, and length was the dorsal distance from the rostral to the caudal end. BW was measured using a Radwag WLC balance (±0.1 g). Finally, the animals were divided into the following categories: large males (LMs; BW > 200 g), medium males (MMs; BW < 200 g), and medium females (MFs; BW < 200 g). These categories were adopted as operational groupings for the feeding trials to assemble experimental groups differing in sex and approximate body size.

*Tanks*

The animals used in the experiments were maintained in two separate tanks connected to the same Recirculating Aquaculture System (RAS) designed for water treatment. The system consisted of a control panel, a circulation pump, a biofilter, a protein skimmer, an ozone generator, a heat exchanger, a UV sterilisation unit, and an aeration system. The first and second tanks (Tanks 1 and 2) were rectangular, with total volumes of 0.75 m^3^ and 1.50 m^3^, respectively. Each tank was divided into several compartments (0.5 m^2^/each): three in Tank 1 and six in Tank 2. The compartments were separated using plastic mesh dividers, allowing the segregation of individuals into different experimental groups.

### 2.1. Feeding Behaviour

**Figure 2:**
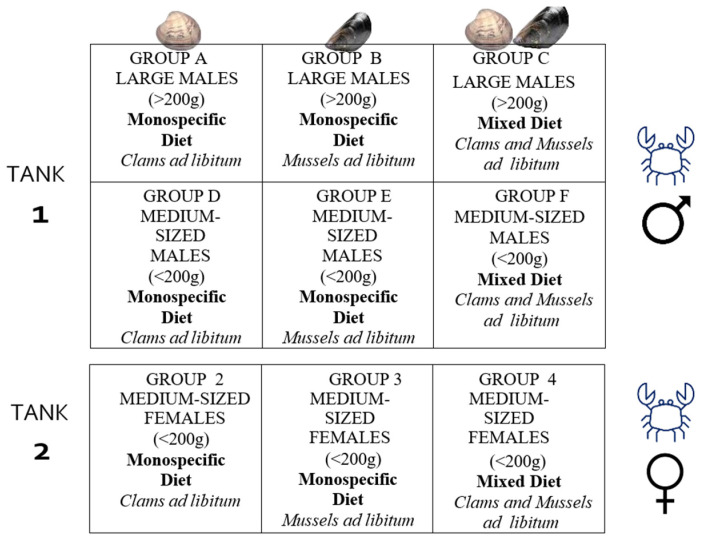
Representation of the arrangement of experimental groups in Tanks 1 and 2 for the “Feeding Behaviour” study.

A total of 56 individuals were used for this experiment, including 31 males and 25 females, grouped by size and sex as shown in Figure 2.

The average density per compartment was 1 ± 0.025 kg of crabs (2.0 kg/m^2^). Groups consisting of MG (compartments A, B, and C) included an average of four individuals, whereas those of MMs (compartments D, E, and F) comprised six to seven individuals. Groups of MFs (compartments G, H, and I) included an average of eight to nine crabs (Figure 2). Each compartment was considered the experimental unit, since crabs were maintained in groups. Food consumption was recorded per compartment, thus avoiding pseudoreplication.

The duration of the trial was 7 days. This study included three experimental treatments:

− A monospecific diet of Manila clams (*Ruditapes philippinarum*) provided ad libitum.
− A monospecific diet of Mediterranean mussels (*Mytilus galloprovincialis*) provided ad libitum.
− A mixed diet of mussels and clams, offered in equal quantities ad libitum.

All treatments were tested on groups which were heterogeneous in size and sex (Figure 2). Both food sources were purchased live from local fish markets and stored in mesh bags in a refrigerator at approximately 4 ± 1 °C until use. Both molluscs were of commercial size, corresponding to the minimum legal landing sizes in the Northern Adriatic: a ≥22 mm shell length for *R. philippinarum* and a ≥50 mm shell length for *M. galloprovincialis*. The same operating procedure was maintained throughout the experimental period.

Each morning, at approximately 09:00 a.m., an operator removed the previous day’s food from the tanks, distinguishing between consumed and unconsumed molluscs and weighing both fractions. From the unconsumed sample, 3–4 clams and/or mussels were randomly collected to determine their total weight and the weight of the edible portion only. After this procedure, at approximately 11:00 a.m., a new daily ration of food was provided. Throughout the trial, water parameters were continuously monitored and recorded to ensure that they remained within the established range: a temperature of 23 ± 1 °C, a dissolved oxygen level of 7.0 ± 0.5 mg L^−1^, pH 8.2 ± 0.1, and a salinity level of 23 ± 1 ppm.

In the feeding preference trials, a two-way analysis of variance (ANOVA) was performed to assess differences in mussel and clam consumption, with sex and individual size included as fixed factors. Model assumptions were evaluated by inspecting residual plots, applying the Shapiro–Wilk test to assess normality of residuals and Levene’s test to verify homogeneity of variances. Differences were considered significant at *p* < 0.05. All analyses were performed in R version 4.5.0.

### 2.2. Prey Handling and Consumption Time

The time of handling and consumption was assessed in 32 MMs. Before the trials, each individual was starved for 24 h to standardise hunger levels. During the experiment, each crab was offered a single prey item, either one mussel or one clam. For each observation, the following behavioural parameters were registered:

− Handling time was defined as the time (s) from the crab’s first contact with the prey using the chelae until the valves were successfully opened. This phase included all attempts to capture and manipulate the bivalve, regardless of whether ingestion occurred.
− Consumption time was defined as the time (s) from valve opening to the complete consumption of the soft tissues or the active rejection of the empty shell.

The duration of shell manipulation and feeding was systematically recorded by a trained operator using a manual stopwatch. Each crab represented an independent replicate, and mean values were calculated for each treatment. All measurements were recorded to the nearest second and subsequently digitised in Microsoft Excel for data management.

The normality and homogeneity of variances for the data were assessed using the Shapiro–Wilk and Levene’s tests, respectively. When required, data were transformed to meet ANOVA assumptions. ANOVA was conducted to evaluate differences in handling and consumption times between mussels and clams. The significance level was set at *p* < 0.05.

### 2.3. Critical Temperature

**Figure 3:**
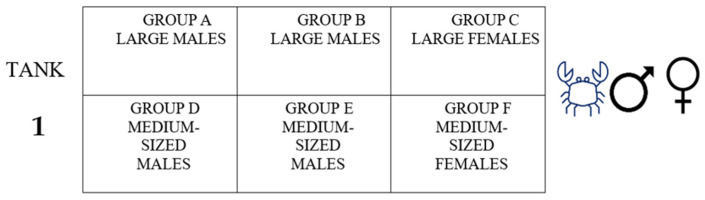
Representation of the distribution of the groups in tank 1 used for the case study.

A total of 31 crabs were used in this study, allocated to experimental compartments according to their sex and body size. The average density per compartment was approximately 1 ± 0.025 kg of crabs, corresponding to 2.0 kg/m^2^. The division of the groups is shown in Figure 3.

The trial included the two following experimental treatments:

− Low temperature: The water temperature decreased by 3 °C every two days.
− High temperature: The water temperature increased by 3 °C every two days.

The water temperature gradually increased or decreased by 3 °C over a 48 h period, with crabs remaining in the tanks to avoid thermal shock.

The operational procedures in the tank followed those described for the first case study. A monospecific diet of mussels was provided ad libitum, which were purchased live from local fish markets and stored in mesh bags in a refrigerator at approximately 4 ± 1 °C until use. Throughout the experimental period, water parameters were kept constant and measured daily: pH 7.8 ± 0.1; a dissolved oxygen level of 7 ± 0.5 mg L^−1^; and a salinity level of 25 ± 0.1 PSU.

The experimental procedures and feeding protocol followed those described in the feeding behaviour trial. The mean food intake of edible portion (g) was compared among eight temperature groups (13,16,19, 22, 25, 28, 31, and 34 °C) using a one-way ANOVA. Before analysis, the assumptions of normality and homogeneity of variances were verified using the Shapiro–Wilk test and Bartlett’s test, respectively. Post hoc pairwise comparisons were performed using Tukey’s test to identify which temperature groups differed significantly. Differences were considered significant at *p* < 0.05. All analyses were performed in R version 4.5.0.

### 2.4. Reproductive Potential

Egg masses were carefully removed from the gonopods of 20 ovigerous females using a scalpel and individually weighed to determine the total egg mass. From each egg mass, ten subsamples (approximately 10 mg each) were collected, weighed using an analytical balance (PRACTUM224-1S; ±0.1 mg precision), and immediately preserved in 10% formalin to arrest development and maintain the original condition at sampling.

Subsamples were examined under a stereomicroscope (Nikon SMZ745T) for egg enumeration. When necessary, aggregated eggs were gently separated to ensure accurate counting. Two counting methods were applied to each subsample: (i) manual counting via direct visual inspection using a manual counter, and (ii) automated counting using the smartphone application CountThings [23] with the “Fish Eggs” template. Automated detection was followed by manual verification and correction when required.

For each subsample, 25 eggs were randomly selected from microscope images for morphometric analysis. Egg diameter was measured using the “Measurements” function in the NIS-Elements software (Nikon, v 5.21.03). The same 25 eggs were also used to determine the developmental stage by visually assessing the relative proportion of yolk and embryo, according to Walker et al.’s procedure [24]. For each female, the following parameters were calculated: mean egg weight (±sd), mean number of eggs per subsample (±sd), and mean number of eggs per gram (NMU). The NMU was estimated as follows:

$$NMU=\frac{mean\;number\;of\;eggs\;per\;subsample}{subsample\;weight\;\left(g\right)}$$

The mean total number of eggs per individual was obtained as follows:

NMU × Total egg mass

The percentage of egg mass relative to total body weight was calculated as:

$$\left(\frac{Total\;egg\;mass}{Body\;weight\;}\right)\times \;100$$

The relationship between biometric and reproductive variables was examined using correlation analyses. The normality of each variable was assessed using the Shapiro–Wilk test. Since only the condition index (CW) met the assumption of normality, Pearson’s correlation coefficients were calculated to evaluate the relationship between CW and absolute fecundity. Statistical significance was set at α = 0.05. All analyses were performed in R version 4.5.0.

### 2.5. Ethics

Blue crabs were handled as per the provisions of the Animal Welfare Committee (COBA) of the University of Bologna, which acknowledged this study (prot. ID 9041). As *C. sapidus* is a decapod crustacean, it does not formally fall within the scope of Directive 2010/63/EU and the related national authorisation requirements.

## 3. Results

### 3.1. Feeding Behaviour

LMs with an average weight of 242 ± 23 g, MMs with an average weight of 155 ± 14 g, and MFs with an average weight of 121 ± 54 g were used for this case study (Table 1).

#### 3.1.1. Effects of a Monospecific Diet (Clams)

For each crab size, the average number of clams consumed, total ingested weight, and edible portion are reported in Table 2. The edible portion mean calculated for clams was 1.74 ± 0.52.

**Figure 4:**
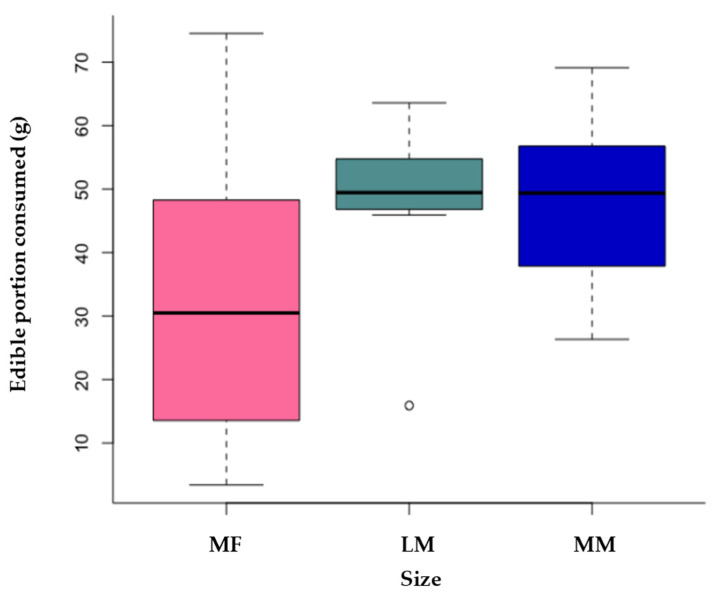
Boxplot showing the amount of edible portion consumed. The *x*-axis indicates animal size class and sex (MF medium females; LM: large males; MM: medium males), while the *y*-axis represents the quantity of edible portion consumed (g).

The results from Table 2 and the boxplot in Figure 4 indicate consistent feeding patterns among size classes, with differences more related to sex than to size. LMs and MMs showed similar mean values in terms of the number of clams consumed, total ingested biomass, and edible portion weight. Their boxplots display comparable medians and relatively narrow interquartile ranges, indicating homogeneous feeding performance within male groups. Although MMs exhibited slightly greater dispersion than LMs, overall variability remained limited. MFs consumed fewer clams on average and showed markedly higher variability, as reflected by the wider interquartile range and broader distribution in the boxplot, suggesting pronounced inter-individual differences in feeding behaviour. Nevertheless, the ANOVA results were not significant for either the sex or the size factor, confirming that the observed differences among groups were not statistically significant.

#### 3.1.2. Effects of a Monospecific Diet (Mussels)

For each crab size, the average number of mussels consumed, total ingested weight, and edible portion are reported in Table 3. The edible portion mean calculated for mussels was 4.50 ± 1.01 g.

**Figure 5:**
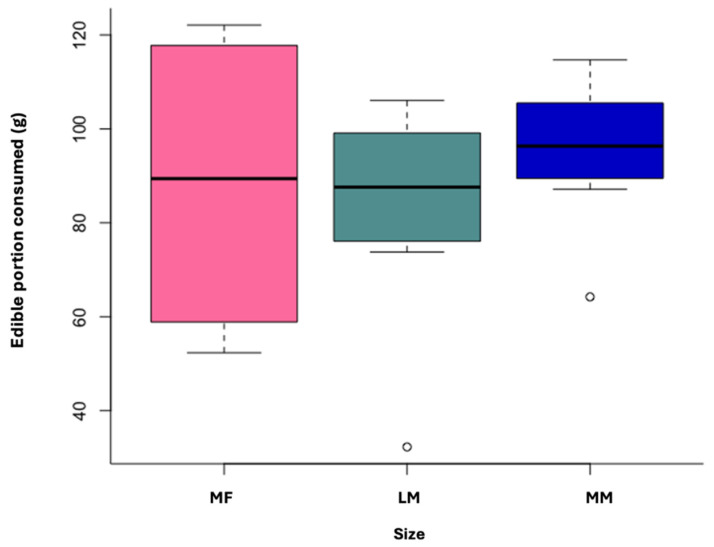
Boxplot showing the amount of edible portion consumed by the three groups during the second trial. The *x*-axis indicates animal size classes (MF: medium females; LM: large males; MM: medium males), while the *y*-axis represents the quantity of edible portion consumed (g).

The results from Table 3 and the boxplot in Figure 5 indicate consistent feeding patterns among crab groups. Differences appeared to be more related to sex than to size.

LMs and MMs showed similar mean values for the number of mussels consumed, total ingested biomass, and edible portion weight. Their boxplots show comparable medians and relatively narrow interquartile ranges, indicating homogeneous feeding performance across male groups. Although MMs showed slightly higher dispersion than LMs, overall variability remained limited. MFs consumed a comparable number of mussels on average but exhibited greater variability, as indicated by the wider interquartile range and broader distribution in the boxplot, suggesting more pronounced inter-individual differences in feeding behaviour. Nevertheless, the ANOVA results were not significant for either sex or size, confirming that the observed differences among groups were not statistically significant.

#### 3.1.3. Use of a Combined Diet (Mussels + Clams)

**Figure 6:**
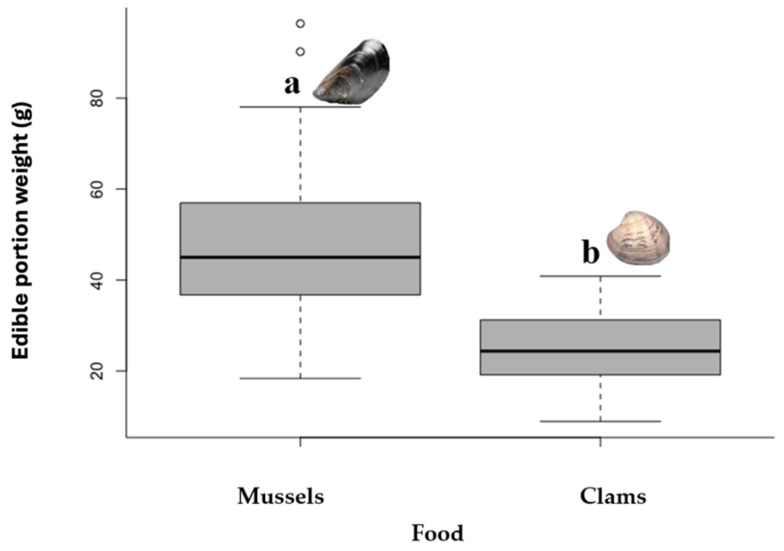
Boxplot showing the amount of edible portion consumed by the three groups during the third trial, comparing mussels and clams. The *x*-axis indicates the type of food, while the *y*-axis represents the quantity of edible portion consumed (g). Different letters (a,b) show significant differences (*p* < 0.05) between the different foods.

The results from Table 4 and the boxplot in Figure 6 indicate that the number of prey items consumed did not markedly differ between clams and mussels across crab groups. All size and sex classes showed comparable predation rates in terms of prey counts, suggesting no strong numerical preference for either bivalve species. However, clear differences emerged when considering the edible portion weight. Although the number of mussels and clams consumed was similar, the edible biomass obtained from mussels was consistently higher and statistically significant (*p* < 0.05), with mean values of 25.21 ± 2.98 g for clams and 47.75 ± 7.98 g for mussels. LMs and MMs exhibited relatively homogeneous feeding behaviour, while MFs showed slightly greater variability, particularly in clam consumption (Table 4).

### 3.2. Prey Handling and Consumption Time

**Figure 7:**
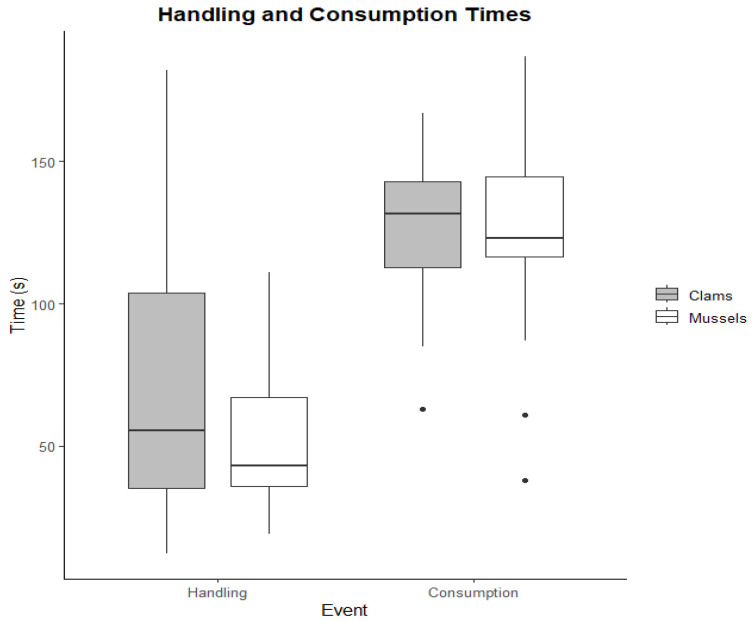
Boxplots of handling and consumption times (s) recorded for medium-sized *Callinectes sapidus* feeding on two bivalve species: clams and mussels.

Figure 7 summarises the valve-opening (handling) and feeding times recorded for clams and mussels. The mean opening time ranged between 1 min 12 s and 1 min 16 s for clams and from 50 s to 1 min 17 s for mussels. The feeding time averaged 1 min 47 s–2 min 02 s for clams and 2 min 08 s–2 min 16 s for mussels. The resulting total handling duration (opening + feeding) ranged from 2 min 50 s to 3 min 04 s for clams and from 3 min 06 s to 3 min 25 s for mussels. Although mussels showed slightly longer feeding and overall processing times, the variability overlapped between prey types, and no statistically significant differences were detected for either handling or consumption time (*p* > 0.05).

### 3.3. Critical Temperature

**Figure 8:**
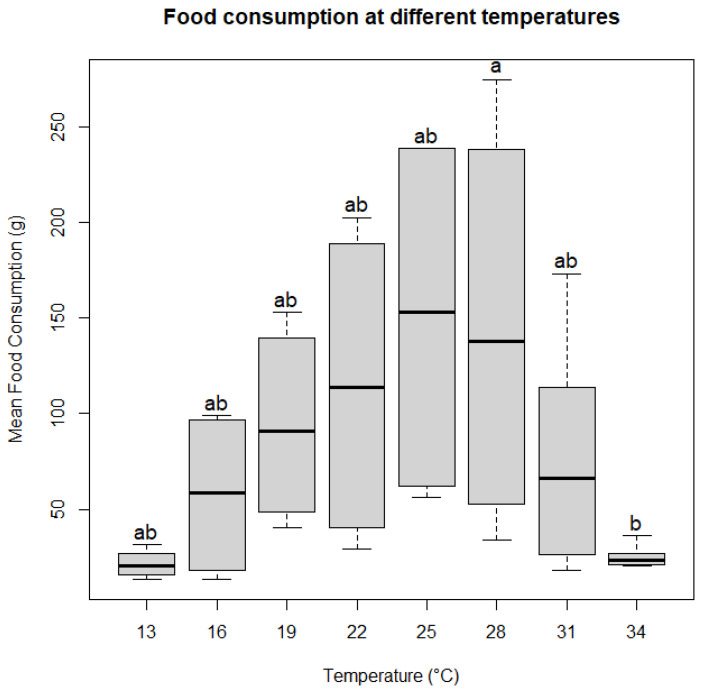
Boxplot showing the variation in mussel consumption with increasing temperature. The *x*-axis represents the tested temperature intervals, while the *y*-axis shows the mean edible weight (g) of mussels consumed. Different letters (a,b) show significant differences (*p* < 0.05) between the different temperatures.

For the second experimental trial, LMs with an average weight of 241 ± 23.36 g, MMs with an average weight of 154 ± 16.88 g, LFs with an average weight of 243.28 ± 20.07 g, and MFs with an average weight of 161.12 ± 20.68 g were used. Food intake significantly varied across temperatures (one-way ANOVA; *p* < 0.05; Figure 8). Consumption increased progressively from 13 °C to 25–28 °C, where the highest median values were recorded, and then sharply declined at 31 °C and 34 °C. Post hoc Tukey’s HSD comparisons (letters in Figure 8) showed that food consumption at 34 °C was significantly lower than at 28 °C. Simultaneously, intermediate temperatures (16–31 °C) did not significantly differ from one another, displaying overlapping statistical groups (ab). The lowest temperatures (13 °C) also showed reduced intake compared with peak values, although differences were less pronounced. Overall, food intake was maximal at intermediate temperatures (25–28 °C) and markedly reduced at both low and high thermal extremes.

### 3.4. Reproductive Potential

**Figure 9:**
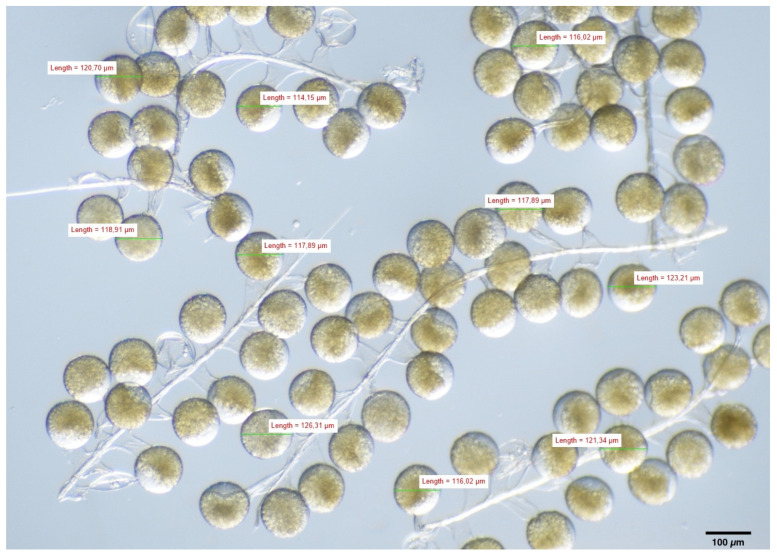
Representative micrograph of *C. sapidus* eggs used for morphometric analysis. Scale bar = 100 µm.

The number of eggs per ovigerous female (absolute fecundity) ranged from 1.0 to 2.7 million eggs. The mean egg diameter was 130.2 ± 8.0 μm. Representative microscopic images of the eggs are shown in Figure 9. The mean absolute fecundity was 1.6 ± 0.5 million eggs, while the relative fecundity (number of eggs per 100 g of body weight) was 0.9 ± 0.3 million. On average, one gram of eggs contained 50,200 ± 7200 eggs. The mean percentage of egg mass relative to total body weight was 20.45 ± 3.1%.

**Figure 10:**
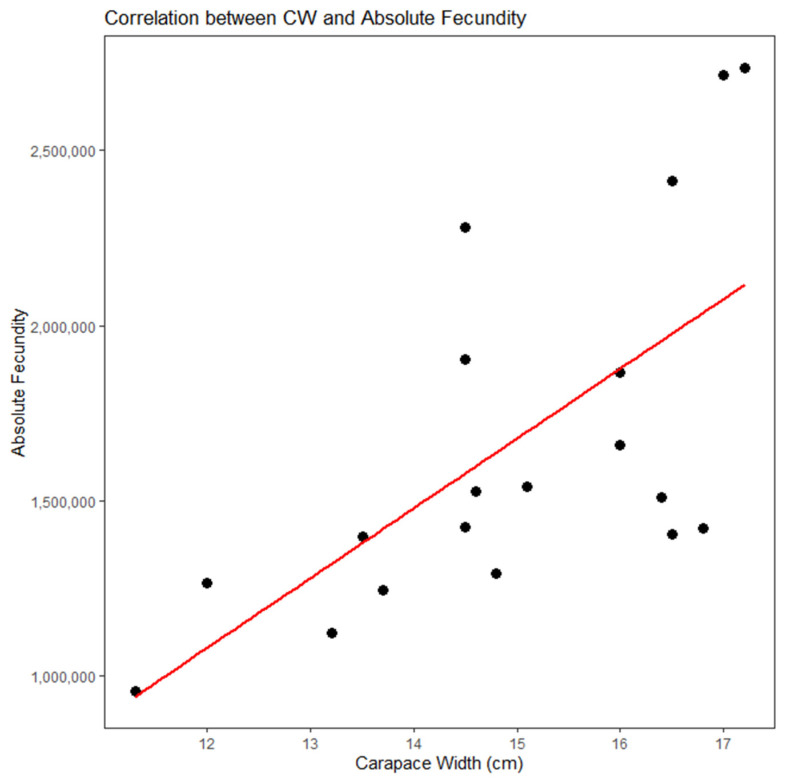
Relationship between carapace width and absolute fecundity. Each point represents an individual, and the line indicates the linear regression fit.

A positive and statistically significant monotonic correlation was detected between absolute fecundity and CW (r = 0.724; *p* < 0.001) (Figure 10), showing that heavier individuals tended to have higher fecundity. The presence of ties does not compromise the test’s robustness.

## 4. Discussion

The consumption of bivalve molluscs by *C. sapidus* represents one of the most relevant aspects for understanding both the trophic role of this species in invaded ecosystems and its potential impacts on shellfish farming activities. In the northern Adriatic context, the applied relevance of this issue is further amplified by the coexistence of highly productive lagoon environments and extensive aquaculture systems dedicated to clam and mussel farming.

In the scientific literature, the blue crab is described as an opportunistic and highly versatile predator capable of exploiting different bivalve species and modulating its feeding behaviour according to prey availability, accessibility, predator size, and prey size. Both classical and recent studies show that predation on bivalves does not depend exclusively on prey species identity, but also on morphological and ecological characteristics that influence prey vulnerability [8,19,25,26].

Within this framework, feeding trials conducted under controlled conditions assume fundamental methodological importance because they allow dietary preference and consumption rates to be isolated while minimising the influence of environmental variables typical of field conditions [8,20,27]. Therefore, these results should be interpreted as estimates of potential predation under conditions of high prey accessibility, rather than as direct quantitative predictions of impact under field or farming conditions.

### 4.1. Feeding Behaviour

In the monospecific feeding trial with *R. philippinarum*, the average edible biomass consumption per unit of predator biomass was broadly similar between large- and medium-sized males, whereas medium-sized females showed greater variability. Since the trial was conducted without sediment, clams were immediately available and did not require searching or extraction from the substrate. Under these conditions, consumption likely depended more on prey manipulation, opening efficiency, and ingestion rate than on accessibility constraints. Accordingly, differences among groups appeared limited, and no statistically significant effects of sex or size were detected. Nevertheless, variability among females suggests that behavioural factors may still play a role [8,19,25,26,27].

Consistently, Boschiero et al. [27] reported that male crabs tended to consume more individuals than females in controlled experiments with Manila clams.

Differences related to crab size and sex tend to emerge more clearly when interacting with prey size and substrate conditions. Such differences may, therefore, be attenuated under standardised conditions and in the absence of sediment [25]. Consistently, Prado et al. [8] reported that bivalve consumption strongly depends on prey type and predator–prey size relationships, rather than on crab body size alone.

When the prey type was switched to mussels, edible biomass consumption increased markedly and remained relatively homogeneous among crab groups. Hence, *Mytilus galloprovincialis* may represent a prey type with higher energetic return and trophic profitability under the experimental conditions adopted. This interpretation is consistent with previous experimental studies showing that mussels can generate higher biomass intake across predator size classes, even when differences in prey number are limited [8,20,28,29]. Cabiddu et al. [20] suggested that the high consumption of mussels may be facilitated by relatively low handling costs. This interpretation aligns with studies addressing mussel selection by blue crabs, which indicate that predation may follow an optimisation strategy based on minimising the manipulation time and maximising the energy intake rate [28], while also being constrained by biomechanical factors such as shell resistance and claw strength [29].

The relative uniformity of consumption among groups may also indicate that mussels likely fell within the handling capacity of all crab size classes within the prey size range offered and under ad libitum conditions. Consequently, the effects of sex and predator size may have been reduced, allowing prey type to emerge as the dominant factor [8].

The mixed-diet trial provides further insight into feeding behaviour. Although the number of clams and mussels consumed did not differ markedly, mussels yielded a higher edible biomass. This suggests that prey use may operate at two complementary levels: attack frequency (number of prey items) and trophic yield (biomass obtained). In this context, a higher numerical consumption of clams may reflect their lower edible fraction per individual, whereas fewer mussels may contribute more substantially to total biomass intake due to a more favourable gain-to-handling cost ratio.

This result is consistent with the literature on blue crab feeding ecology, according to which prey choice reflects a trade-off between energetic return and manipulation costs [26,29]. Cabiddu et al. [20] similarly observed that numerical prey preference may diverge from ingested biomass, emphasising the importance of distinguishing between prey counts and trophic yield when assessing ecological or aquaculture impacts.

In lagoon environments, predation may be further modulated by the physical accessibility of resources. Clams represent a benthic resource which is directly available along the normal foraging pathway of blue crabs. In contrast, mussels cultured in suspension (e.g., on ropes, longlines, or similar structures) may be less accessible, requiring additional access costs such as swimming or climbing along structures, potentially increasing the probability of disturbance or interruption of the predatory event.

These considerations highlight the need to interpret no-substrate feeding trials as estimates of potential consumption under conditions of maximum prey accessibility. Therefore, the present results should be interpreted cautiously when extrapolating to natural or farming systems, where predation outcomes are also influenced by sediment characteristics, prey burial behaviour, habitat structure, and farming practices.

Within this context, our findings should be considered as biologically informative estimates of short-term feeding performance rather than direct predictors of field impact. Nonetheless, they support the view that trophic flexibility and the ability to exploit prey with different energetic returns may contribute to the ecological success of *C. sapidus* in invaded systems [8,20]. Studies conducted in the Po Delta have documented significant losses in clam production and an increasing need for structured monitoring, containment, and adaptive management of this invasive species [22,30,31].

Within this context, this study provides a useful contribution by linking experimental consumption and preference patterns with a concrete management problem, providing biological insights that support trophic risk assessment in lagoon aquaculture systems.

### 4.2. Prey Handling and Consumption Time

Separately measuring the time required to open the valves and the time required to consume the tissues allows for a realistic description of the temporal cost associated with prey processing, thus providing insight into prey choice and potential impacts on natural beds and aquaculture systems, as reported by Ebersole and Kennedy [26].

In this study, clams tended to require slightly longer valve-opening times, whereas the subsequent consumption phase was generally shorter. In contrast, mussels showed comparable or occasionally faster opening times but tended to require longer tissue consumption phases. Consequently, total handling durations overlapped considerably between prey types, and no statistically significant differences were detected for either opening or consumption time.

This “crossed pattern” suggests that prey-specific differences may emerge in distinct components of the predation sequence and that compensatory effects between opening and post-opening phases can reduce differences in the total processing time. A similar interpretation was proposed by Hughes and Seed [32], who highlighted that the total handling time reflects the sum of multiple behavioural components, which may vary independently and partially compensate for each other.

Cabiddu et al. provided a relevant comparison in the Mediterranean context [20] for *C. sapidus*, distinguishing between a manipulation/breaking phase (capture–crushing) and a consumption phase (crushing–release of shell fragments). In their study, manipulation was faster for *Mytilus galloprovincialis* than for *Ruditapes philippinarum* on average, whereas consumption times were longer and highly variable, with no significant differences in total handling time or consumption time. This suggests that the post-breaking phase may be the most variable and may blur the separation between prey species.

This pattern is consistent with the idea that efficient initial shell breakage does not necessarily translate into rapid consumption, because the post-opening phase includes tissue extraction and handling of shell fragments and is, therefore, sensitive to individual differences and feeding techniques, as discussed by Elner [33] and Aronhime and Brown [28].

Finally, other studies have indicated that the magnitude of handling times depends on both the mechanical difficulty of the prey and the temporal interval considered. Ebersole and Kennedy [26], using an inclusive definition extending until shell abandonment for ≥60 s, reported an average handling time of 1070 s for *C. sapidus* feeding on Mya arenaria, illustrating how more-resistant prey and broader definitions of handling time can dramatically increase measured durations.

Overall, the breakdown of processing times indicates that clams tend to concentrate a larger proportion of the cost in the opening phase and less in the consumption phase, whereas mussels show comparable or faster opening but longer consumption. The absence of significant differences is, therefore, consistent with the framework proposed by Hughes and Seed [32] and with the Mediterranean results of Cabiddu et al. [20], where the post-opening component often represents the most variable phase even when mean trends differ.

### 4.3. Critical Temperature

Temperature represents one of the main environmental parameters regulating the trophic performance of *C. sapidus.* It simultaneously influences metabolism, locomotor activity, and prey handling efficiency, thereby modulating food intake along a thermal gradient [9,34,35]. In ectothermic species, these processes typically result in a non-linear response of food intake, with a maximum performance at intermediate temperatures and a reduction at both extremes due to functional limitations at low temperatures and physiological stress above the optimal thermal range [9,34]. In this study, food intake followed a unimodal pattern along the thermal gradient, with peak consumption observed at 25–28 °C and reduced intake at both lower and higher temperatures. This suggests that feeding performance may be enhanced within an intermediate thermal window and constrained at thermal extremes, following the general response expected in ectothermic predators [9,34,35].

The observed reduction in consumption at low temperatures is consistent with a strong limitation of trophic activity under cold conditions. Leffler [34] described that crabs may enter a state of cold-induced inactivity (“cold stupor”) at 13 °C, characterised by reduced locomotion and responsiveness. In this study, consumption at 13 °C was markedly reduced compared with intermediate temperatures, suggesting a substantial limitation of feeding activity. Furthermore, Brylawski and Miller [36] identified a torpor temperature of approximately 10.8 °C, below which activity is strongly reduced and growth is halted.

At the upper end of the thermal gradient, the reduction in consumption beyond 28 °C suggests entry into a supra-optimal range, where physiological costs may increase and feeding efficiency may decline. This pattern is consistent with previous studies reporting optimal performance at intermediate temperatures and reduced activity at higher values [9,35].

However, these results should be interpreted with caution as the experimental design involved temperature changes of 3 °C every two days, likely reflecting short-term responses rather than full physiological acclimation [9,34,35,36].

From an ecological perspective, the peak in feeding activity at 25–28 °C suggests that predation pressure may increase when environmental temperatures approach this range. Nevertheless, extrapolation to natural or aquaculture systems should remain cautious, as field outcomes depend on additional factors, such as prey accessibility and habitat structure [7,8,9,22].

Overall, these findings support the hypothesis that temperature influences trophic performance in *C. sapidus*, although the results should be interpreted as indicative of short-term responses under controlled conditions rather than long-term ecological dynamics [9].

### 4.4. Reproductive Potential

The reproductive results confirm that *C. sapidus* is characterised by high fecundity, with ovigerous females producing large numbers of eggs and showing a positive relationship between fecundity and carapace width. This supports the well-established role of maternal size as a key determinant of reproductive output, and highlights the importance of reproductive capacity in sustaining population persistence and expansion in both native and invaded ranges [10,11,12,13].

The fecundity values observed in this study broadly overlap with those reported in other Mediterranean populations, including Sicily [6] and the eastern Mediterranean [13], confirming that high reproductive output is maintained across invaded areas. Differences among regions likely reflect variability in environmental conditions, sampling periods, and population structure.

Comparisons with the native range indicate that higher fecundity values may occur, particularly in larger individuals or under favourable seasonal conditions. For example, studies from the United States have reported substantial variability linked to reproductive stage and seasonality, with primiparous females showing marked differences in egg production across reproductive periods [10,11]. This suggests that discrepancies among studies are more likely related to biological and ecological factors than to a reduction in reproductive capacity in invaded populations. Overall, the results support the view that the species maintains a high reproductive potential in the northern Adriatic. When considered together with feeding performance, this high fecundity contributes to a combination of traits that may enhance population persistence, recruitment, and spread in newly colonised ecosystems [6,10,11,13].

## 5. Conclusions

This study shows that *Callinectes sapidus* combines trophic flexibility and high reproductive output, traits that likely contribute to its invasive success in the northern Adriatic. Under controlled conditions, blue crabs efficiently consumed both clams and mussels; however, mussels yielded a greater edible biomass, indicating that trophic impact should be interpreted in terms of prey number as well as prey profitability and processing costs. Feeding activity was highest at intermediate temperatures, suggesting that environmental conditions may further modulate predation pressure.

The reproductive analysis confirmed high fecundity in ovigerous females, with larger individuals producing more eggs. Taken together, these trophic and reproductive traits provide a coherent biological explanation for the persistence and expansion of the species in recently colonised Mediterranean environments.

From an applied perspective, this study provides useful biological information for assessing the possible implications of blue crab invasion for shellfish aquaculture in the northern Adriatic. However, because the feeding experiments were conducted under controlled laboratory conditions, further field-based or semi-natural studies are needed to evaluate how habitat structure, sediment, prey accessibility, and farming systems influence the actual impacts of predation.

## Figures and Tables

**Table 1:** Morphometric measurements for each size class and sex of the animals used in the feeding preferences trial. MFs: medium females; LMs: large males; MMs: medium males.

Parameter	LMs	MMs	MFs
Weight (g)	242 ± 23	155 ± 14	121 ± 54
Width (cm)	15 ± 0.75	13.14 ± 0.47	12.76 ± 2.54
Length (cm)	7.36 ± 0.49	6.32 ± 0.30	5.77 ± 0.89

**Table 2:** Number of individuals and biomass, as well as the number, total weight, and edible portion weight of clams consumed by the MFs (medium females), LMs (large males), and MMs (medium males) during the first trial. BW: body weight.

Size	N. Animals	Consumed Clams (n)	Total Clams Weight (g)	Edible Portion Weight (g/Kg Crab BW)
LMs	4	26.86 ± 8.73	345.70 ± 112.32	47.43 ± 15.41
MMs	7	29 ± 9.20	347.82 ± 110.36	47.72 ± 15.14
MFs	9	19.57 ± 15.12	241.57 ± 186.63	33.14 ± 25.60

**Table 3:** Number of individuals and biomass, as well as the number, total weight, and edible portion weight of mussels consumed by MFs: medium females; LMs: large males; MMs: medium males during the second trial.

Size	N. Animals	Consumed Mussels (n)	Total Mussels Weight (g)	Edible Portion Weight (g/kg Crab BW)
LMs	4	17.86 ± 5.49	287.14 ± 90.06	82.33 ± 25.31
MMs	6	20.71 ± 3.77	338,12 ± 61.59	95.02 ± 17.31
MFs	8	20.25 ± 7.93	314.21 ± 123.08	88.30 ± 34.59

**Table 4:** Number of individuals and biomass, as well as the number, total weight, and edible portion weight of clams consumed by MFs (medium females), LMs (large males), and MMs (medium males) during the second trial.

Size	N. Animals	Consumed Clams (n)	Total Clams Weight (g)	Edible Portion Weight (g/Kg Crab BW)	Food
LMs	4	12.29 ± 5.88	152.90 ± 73.17	21.80 ± 10.44	Clams
LMs	4	11.57 ± 5.86	189.81 ± 96.05	53.12 ± 26.88	Mussels
MMs	6	14.43 ± 4.20	185.72 ± 54.03	26.49 ± 7.71	Clams
MMs	6	10.86 ± 4.45	184.20 ± 75.51	51.55 ± 21.13	Mussels
MFs	8	15.71 ± 4.11	191.54 ± 50.11	27.34 ± 7.15	Clams
MFs	8	8.57 ± 2.64	137.71 ± 42.36	38.57 ± 11.87	Mussels

## Data Availability

The data presented in this study are available on request from the corresponding author.

## References

[1] Streftaris N., Zenetos A. (2006). Alien Marine Species in the Mediterranean—The 100 ‘Worst Invasives’ and Their Impact. Medit. Mar. Sci..

[2] Manfrin C., Comisso G., Dall’Asta A., Bettoso N., Chung J.S. (2016). The Return of the Blue Crab, *Callinectes sapidus* Rathbun, 1896, after 70 Years from Its First Appearance in the Gulf of Trieste, Northern Adriatic Sea, Italy (Decapoda: Portunidae). Check List.

[3] Mancinelli G., Chainho P., Cilenti L., Falco S., Kapiris K., Katselis G., Ribeiro F. (2017). The Atlantic Blue Crab *Callinectes sapidus* in Southern European Coastal Waters: Distribution, Impact and Prospective Invasion Management Strategies. Mar. Pollut. Bull..

[4] Falsone F., Scannella D., Geraci M.L., Vitale S., Sardo G., Fiorentino F. (2020). Further Records of *Callinectes sapidus* (Rathbun, 1896) (Decapoda, Brachyura, Portunidae) in the Strait of Sicily. Mar. Biodivers. Rec..

[5] Shauer M., Zangaro F., Specchia V., Pinna M. (2025). Investigating Invasion Patterns of *Callinectes sapidus* and the Relation with Research Effort and Climate Change in the Mediterranean Sea. Sci. Rep..

[6] Marchessaux G., Gjoni V., Sarà G. (2023). Environmental Drivers of Size-Based Population Structure, Sexual Maturity and Fecundity: A Study of the Invasive Blue Crab *Callinectes sapidus* (Rathbun, 1896) in the Mediterranean Sea. PLoS ONE.

[7] Cannarozzi L., Paoli C., Vassallo P., Cilenti L., Bevilacqua S., Lago N., Scirocco T., Rigo I. (2023). Donor-Side and User-Side Evaluation of the Atlantic Blue Crab Invasion on a Mediterranean Lagoon. Mar. Pollut. Bull..

[8] Prado P., Peñas A., Ibáñez C., Cabanes P., Jornet L., Álvarez N., Caiola N. (2020). Prey Size and Species Preferences in the Invasive Blue Crab, *Callinectes sapidus*: Potential Effects in Marine and Freshwater Ecosystems. Estuar. Coast. Shelf Sci..

[9] Marchessaux G., Bosch-Belmar M., Cilenti L., Lago N., Mangano M.C., Marsiglia N., Sarà G. (2022). The Invasive Blue Crab *Callinectes sapidus* Thermal Response: Predicting Metabolic Suitability Maps under Future Warming Mediterranean Scenarios. Front. Mar. Sci..

[10] Prager M.H., McConaugha J.R., Jones C.M., Geer P.J. (1990). Fecundity of Blue Crab, *Callinectes sapidus*, in Chesapeake Bay: Biological, Statistical and Management Considerations. Bull. Mar. Sci..

[11] Graham D.J., Fulford R., Biesiot P., Perry H. (2012). Fecundity and Egg Diameter of Primiparous and Multiparous Blue Crab *Callinectes sapidus* (Brachyura: Portunidae) in Mississippi Waters. J. Crustac. Biol..

[12] Schneider A.K., Shields J.D., Fabrizio M.C., Lipcius R.N. (2024). Spawning History, Fecundity, and Potential Sperm Limitation of Female Blue Crabs in Chesapeake Bay. Fish. Res..

[13] Kevrekidis K., Kevrekidis T., Chitinroglou C.C., Avramoglou K., Keisaris S., Fryganiotis K., Apostologamvrou C., Roditi K., Voulgaris K., Varkoulis A. (2024). Reproductive Biology of the Invasive Blue Crab *Callinectes sapidus* in the Thermaikos Gulf (Northwest Aegean Sea, Eastern Mediterranean): Identifying Key Information for an Effective Population Management Policy. J. Mar. Sci. Eng..

[14] Ortega-Jiménez E., Cuesta J.A., Laiz I., González-Ortegón E. (2024). Diet of the Invasive Atlantic Blue Crab *Callinectes sapidus* Rathbun, 1896 (Decapoda, Portunidae) in the Guadalquivir Estuary (Spain). Estuar. Coast..

[15] Cerri J., Chiesa S., Bolognini L., Mancinelli G., Grati F., Dragičević B., Dulčic J., Azzurro E. (2020). Using Online Questionnaires to Assess Marine Bio-Invasions: A Demonstration with Recreational Fishers and the Atlantic Blue Crab *Callinectes sapidus* (Rathbun, 1986) along Three Mediterranean Countries. Mar. Pollut. Bull..

[16] Prado P., Ibáñez C., Chen L., Caiola N. (2022). Feeding Habits and Short-Term Mobility Patterns of Blue Crab, *Callinectes sapidus*, Across Invaded Habitats of the Ebro Delta Subjected to Contrasting Salinity. Estuar. Coast..

[17] Khamassi F., Rjiba Bahri W., Mnari Bhouri A., Chaffai A., Soufi Kechaou E., Ghanem R., Ben Souissi J. (2022). Biochemical Composition, Nutritional Value and Socio-Economic Impacts of the Invasive Crab *Callinectes sapidus* Rathbun, 1896 in Central Mediterranean Sea. Medit. Mar. Sci..

[18] Eggleston D.B. (1990). Functional Responses of Blue Crabs *Callinectes sapidus* Rathbun Feeding on Juvenile Oysters *Crassostrea virginica* (Gmelin): Effects of Predator Sex and Size, and Prey Size. J. Exp. Mar. Bio. Ecol..

[19] Prado P., Gairin I., Falco S. (2024). Effect of Bivalves’ Sand Burial Capacity on Predation in the Invasive Blue Crab, *Callinectes sapidus*. J. Mar. Sci. Eng..

[20] Cabiddu S., Addis P., Palmas F., Pusceddu A., Solari P., Pasquini V. (2025). Feeding Behaviour and Preference of the Invasive Blue Crab (*Callinectes sapidus* Rathbun, 1896) for Mediterranean Native Bivalves in Mesocosm. Hydrobiologia.

[21] Tiralongo F., Nota A., Pasquale C.D., Muccio E., Felici A. (2024). Trophic Interactions of *Callinectes sapidus* (Blue Crab) in Vendicari Nature Reserve (Central Mediterranean, Ionian Sea) and First Record of *Penaeus aztecus* (Brown Shrimp). Diversity.

[22] Gavioli A., Mancinelli G., Turolla E., Lanzoni M., Paesanti V., Soana E., Eggleston D.B., Christian R.R., Castaldelli G. (2025). Impacts of the Invasive Blue Crab *Callinectes sapidus* on Small-Scale Fisheries in a Mediterranean Lagoon Using Fishery Landing Data. Sci. Total Environ..

[23] Dynamic Ventures Inc. (2024). CountThings.

[24] Walker A., Ando S., Smith G.D., Lee R.F. (2006). The Utilization of Lipovitellin during Blue Crab (*Callinectes sapidus*) Embryogenesis. Comp. Biochem. Physiol. B Biochem. Mol. Biol..

[25] Arnold W.S. (1984). The Effects of Prey Size, Predator Size, and Sediment Composition on the Rate of Predation of the Blue Crab, *Callinectes sapidus* Rathbun, on the Hard Clam, *Mercenaria Mercenaria* (Linné). J. Exp. Mar. Biol. Ecol..

[26] Ebersole E.L., Kennedy V.S. (1995). Prey Preferences of Blue Crabs *Callinectes sapidus* Feeding on Three Bivalve Species. Mar. Ecol. Prog. Ser..

[27] Boschiero M., Facca C., Cavraro F., Tonolli M., Malavasi S., Franzoi P. (2025). Feeding Strategies of the Invasive Blue Crab (*Callinectes sapidus*) on Manila Clam (*Ruditapes Philippinarum*): Implications for Aquaculture. Estuar. Coast. Shelf Sci..

[28] Aronhime B.R., Brown K.M. (2009). The Roles of Profit and Claw Strength in Determining Mussel Size Selection by Crabs. J. Exp. Mar. Biol. Ecol..

[29] Seed R., Hughes R.N. (1997). Chelal Characteristics and Foraging Behaviour of the Blue Crab Rathbun. Estuar. Coast. Shelf Sci..

[30] Chiesa S., Petochi T., Brusà R.B., Raicevich S., Cacciatore F., Franceschini G., Antonini C., Vallini C., Bernarello V., Oselladore F. (2025). Impacts of the Blue Crab Invasion on Manila Clam Aquaculture in Po Delta Coastal Lagoons (Northern Adriatic Sea, Italy). Estuar. Coast. Shelf Sci..

[31] Gaglio M., Gavioli A., Turolla E., Lanzoni M., Castaldelli G. (2025). The Costs of an Invasion: How the Blue Crab Impaired Ecosystem Services in the Most Productive Lagoon of Northwestern Adriatic. Sci. Total Environ..

[32] Hughes R., Seed R. (1981). Size Selection of Mussels by the Blue Crab *Callinectes sapidus*: Energy Maximizer or Time Minimizer?. Mar. Ecol. Prog. Ser..

[33] Elner R.W. (1978). The Mechanics of Predation by the Shore Crab, *Carcinus maenas* (L.), on the Edible Mussel, *Mytilus edulis* L.. Oecologia.

[34] Leffler C.W. (1972). Some Effects of Temperature on the Growth and Metabolic Rate of Juvenile Blue Crabs, *Callinectes sapidus,* in the Laboratory. Mar. Biol..

[35] Glandon H.L., Miller T.J. (2017). No Effect of High pCO2 on Juvenile Blue Crab, *Callinectes sapidus*, Growth and Consumption despite Positive Responses to Concurrent Warming. ICES J. Mar. Sci..

[36] Brylawski B.J., Miller T.J. (2006). Temperature-Dependent Growth of the Blue Crab (*Callinectes sapidus*): A Molt Process Approach. Can. J. Fish. Aquat. Sci..

